# Intraoperative continuous cerebral microcirculation measurement in patients with aneurysmal subarachnoid hemorrhage: preliminary data on the early administration of magnesium sulfate

**DOI:** 10.1186/s12871-017-0435-y

**Published:** 2017-10-17

**Authors:** Bjoern Sommer, Cornelia S. Weidinger, Dennis Wolf, Michael Buchfelder, Hubert Schmitt

**Affiliations:** 1 Department of Neurosurgery, University Hospital Erlangen, Schwabachanlage 6, 91054 Erlangen, Germany; 2 Department of Anesthesiology, University Hospital Erlangen, Krankenhausstraße 12, 91054 Erlangen, Germany; 3 Department of Cardiology and Angiology I, University Heart Center Freiburg, Hugstetter Straße 55, 79106 Freiburg, Germany; 4 Department of Neurosurgery, Paracelsus-Klinik Osnabrück, Am Natruper Holz 69, 49076 Osnabrück, Germany

**Keywords:** Cerebral blood flow, Cerebral vasospasm, Intraoperative monitoring, Neurosurgery, Ruptured aneurysm

## Abstract

**Background:**

In patients with subarachnoid hemorrhage (SAH), vasospasm remains one of the major complications. The application of intravenous magnesium sulfate (MgSO_4_) has been under discussion to prevent cerebral ischemia. Our aim was to examine the impact of early MgSO_4_ administration on local cerebral microcirculation during microsurgical clipping of SAH-related aneurysms.

**Methods:**

The non-invasive laser-Doppler spectrophotometry system “Oxygen-to-See (O2C)” was used in 14 consecutive patients (11 female, 3 male, median age 56.5±9.7 yrs) with aneurysmatic SAH. A subdural probe measured capillary venous oxygenation (SO2), relative hemoglobin content (rHb), blood cell velocity (velo) and blood flow (flow) in 7 mm tissue depth. Data samples were recorded as baseline immediately before intraoperative application of MgSO_4_ 10% 50 mg/kg body weight and 10 min thereafter. The continuous MgSO_4_ infusion rate depended on blood pressure (mean arterial pressure > 60-65 mmHg) and lasted a maximum of 60 min.

**Results:**

MgSO_4_ was administered 2.8 (min. 1.6, max. 15.5) hours after onset of symptoms. Median flow increased significantly by 20.8% (5–68%, *p* = 0.001). Velo increased 4.9% (1–17%), rHb decreased 1.5% (3–34%) and SO2 decreased 9.4% (8–38%) by trend compared to the baseline values. FiO_2_ correlated positively with velo (r_s_ = 0.712, *p* = 0.004), whereas arterial HCO_3_ correlated negatively with SO2 (r_s_ = −0.599, *p* = 0.024). Of 14 patients, 2 had symptomatic vasospasm.

**Conclusions:**

Our data suggest an increased cerebral blood flow after early intraoperative administration of MgSO_4_ in patients with SAH. Using a non-invasive laser-Doppler spectrophotometry system, this technique is feasible for continuous real-time monitoring of cerebral microcirculation.

**Trial registration:**

DRKS (German Clinical Trial Registry), DRKS00013047, retrospectively registered on September 21^st^, 2017.

## Background

Subarachnoid hemorrhage (SAH) caused by a ruptured aneurysm occurs in about 700.000 individuals per year worldwide with an approximate incidence of 9 per 100.000 person years [1]. This adds up about 36.000 SAH cases per year in the European Community [2, 3]. After aneurysmal subarachnoid hemorrhage, high morbidity rates in approximately 30% of patients with severe permanent disability due to cerebral infarction are reported [4, 5]. One of the most important factors associated with poor clinical outcome is early or delayed cerebral vasospasm (CVS) [5–7]. CVS is a biphasic process, with an acute phase beginning 10 min to 4 h after the initial bleeding event and a delayed, chronic phase with symptoms resulting from ischemia typically starting 3 to 14 days thereafter [1, 8].

A variety of treatment options aiming at the relaxation of smooth muscle in cerebral arteries to prevent CVS and avoid infarctions have been reported, including the application of calcium antagonists, statins, or endothelin receptor antagonists [8]. As interpretations of the results still remain controversial, no standard treatment for CVS has yet been established. Magnesium, which physiologically acts as a calcium antagonist, has also been suggested as a potential therapeutic agent for CVS.

Unfortunately, several randomized controlled trials and meta-analysis could not clearly show the positive benefit of magnesium therapy. The MASH trial suggested a reduction of delayed cerebral ischemia and related poor clinical outcome [9]. However, the IMASH and MASH-2 trials compared magnesium sulfate with placebo and both failed to show a beneficial effect of magnesium on the clinical condition [10, 11].

There are several possible explanations for the lack of evidence in pooled studies. In most randomized trials, time from SAH to magnesium administration has been >24 h after bleeding, and no homogenous timing of administration or dosing schedules were applied [12–14]. The most recent meta-analysis concerning early administration of magnesium within 24 h showed no beneficial effect on poor outcome or delayed ischemic deficit, as well [15]. However, only 83 of 1981 included patients were treated within 6 h after SAH onset. Another aspect is the insufficient concentration of magnesium in cerebrospinal fluid (CSF) to evoke neuroprotective effects [7, 10]. Even though another meta-analysis acknowledges timing of initiation, inappropriate dosing or duration of therapy as potential factors which could hamper positive effects of magnesium on clinical outcome, a treatment effect could not be confirmed [16]. Using a novel non-invasive laser-Doppler spectrophotometry system, local cerebral microcirculation can be continuously measured in real-time mode during neurosurgical procedures [17–19]. Thus, we wanted to evaluate the influence of early magnesium sulfate administration on local blood perfusion in SAH patients undergoing aneurysm surgery.

## Methods

Patient recruitment started in January 2014 at the Department of Neurosurgery, University Hospital Erlangen. This study complied with the laws of the Federal Republic of Germany and followed the Declaration of Helsinki. It was approved by the Ethical Board of the Friedrich-Alexander University of Erlangen-Nuremberg (reference number 4570). Written informed consent was obtained from all subjects or a legal surrogate.

Inclusion criteria were: a) age 18–85 years, b) supratentorial craniotomy, c) microsurgical clipping of an intracerebral aneurysm causing SAH, and d) craniotomy size >3 cm in diameter. Exclusion criteria were a) aneurysm of the posterior circulation, b) decompensated renal or liver insufficiency, c) acute coronary syndrome, d) severe comorbidity (modified rankin skale (mRS) ≥3), e) pregnancy.

### Experimental setup

#### Surgery

After verification of SAH and identification of the intracerebral aneurysm by digital subtraction angiography or CT angiography, patients were transferred to the operating room immediately. Every patient received an external ventricle drainage prior to craniotomy. Also, 2 g cefazolin was given as an intravenous antibiotic prophylaxis.

After fixing the skull in the Mayfield clamp, we set up the SEP monitoring (Nicolet Endeavor CR or Viking IV-P, Viasis Healthcare, Pennsylvania, U.S.A.). Bilateral median nerve stimulation at the wrist or posterior tibial nerve stimulation at the ankle was applied (intensity 20–25 mA, duration 200 μs, rate 5.1 Hz, Table 1). Subdermal recording electrodes were placed on the three scalp sides (C3’, C4’, Cz’) according to the international 10–20 system, with a reference electrode at Fz. Settings of the data processing were: bandpass filter 30 to 300 Hz, sensitivity 7 μV, 200 sweep averages. After verification of correct SEP-monitoring, surgery was started.

**Table 1:** Patient characteristics, monitoring and functional outcome

Patient No.	Age range	Comorbidity	SAB grade (WFNS)	Aneurysm location	Side	Probe placement	SEP monitoring	Pathological SEP?	Vasospasm/related neurological deficite?	mRS at discharge	mRS at 12 months
1	3	AH	V	AcoA	L	temporal	Posterior tibial nerve	yes	no	5	4
2	2	AH	II	ACA	R	temporal	n.a.	n.a.	yes/no	1	1
3	2	AH	III	AcoA	L	frontal	Posterior tibial nerve	yes	yes/no	2	1
4	1	none	II	ACA	R	temporal	Posterior tibial nerve	no	yes/no	0	0
5	3	none	IV	AcoA	R	frontal	Posterior tibial nerve	yes	no	2	1
6	2	AH	II	MCA	R	temporal	Median nerve	no	no	2	2
7	1	none	IV	MCA	L	temporal	Median nerve	yes	no	3	3
8	3	AH	II	AcoA	L	temporal	Posterior tibial nerve	no	no	1	0
9	3	AH	V	AcoA	L	frontal	Posterior tibial nerve	yes	yes/yes	6	6
10	2	AH	III	MCA	R	temporal	Median nerve	yes	yes/no	4	4
11	3	AH	IV	MCA	R	temporal	Median nerve	no	yes/no	1	0
12	4	AH, CAD, HI	V	MCA	L	temporal	n.a.	n.a.	no	6	6
13	2	AH	II	MCA	R	frontal	Median nerve	yes	yes/no	1	1
14	1	AH	III	MCA	L	temporal	Median nerve	yes	yes/yes	3	3

Age range (years) 1 = 40–49, 2 = 50–59, 3 = 60–69, 4 = 80–85. *AcoA* anterior communicating artery, *ACA* anterior cerebral artery, *MCA* middle cerebral artery, *AH* arterial hypertension, *CAD* coronary artery disease, *HI* heart insufficiency, *mRS* modified Rankin Scale, *WFNS* World Federation of Neurosurgical Societies

#### Intraoperative vascular neuromonitoring

The measurement technique of the laser-Doppler spectrophotometry system “O2C” has been described in detail elsewhere [19, 20]. In summary, backscattering tissue spectrophotometry is used to assess local cerebral capillary-venous oxygen saturation (SO2) as well as blood volume status by calculating the relative hemoglobin content (rHb). At the same time, the integrated laser-Doppler flowmeter measures the relative velocity of moving blood cells (velo) and relative blood flow (flow) by using the Doppler frequency shift formula. The spectrophotometry system has a sampling rate of 2 Hz, the laser-Doppler flowmeter of 40 Hz. Both measurement techniques are combined in the O2C flat probe “LFX25” used during this investigation. As the microcirculatory parameters have not been validated for human brain tissue, yet, *SO2*, *rHb*, *velo* and *flow* present pseudo-quantitative variables. Thus, the scaling is given either in % for SO2, or arbitrary units (AU) for the other three parameters. Validity as well as reliability of the obtained variables was tested and proved sufficient in several studies, including invasive cerebral blood flow measurements with colored microspheres in animals [17–20].

**Fig. 1:**
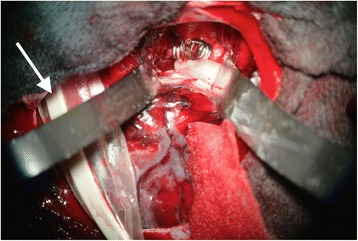
View through the operating microscope in patient No. 14 with SAH grade 3 (WFNS) caused by a ruptured aneurysm of the left middle cerebral artery. The subdural probe (white arrow) is placed on the cortex of the frontal lobe away from the surgical preparation site. The two aneurysm clips can be seen right between the self-retaining retractors

After microsurgical opening of the dura, the O2C probe was carefully inserted subdurally and placed at the distal supply territory of the parent aneurysmatic vessel under continuous irrigation to avoid damage of brain tissue (Fig. 1). The probe position was dependent on the degree of brain swelling, hindrance due to brain structures such as bridging veins or the surgical manoeuvrability (Table 1). After ambient light correction and visual verification of stable parameters, which lasted approximately 30 s, continuous real-time monitoring was started. The baseline measurement sample included a time span of 60 s, which was recorded when all parameters remained stable. Then, we began the application of MgSO_4_ and 10 min thereafter, another data sample of 60 s was taken. The neurosurgeon was blinded to the exact beginning of the MgSO_4_ administration and the result of the vascular neuromonitoring parameters obtained by the O2C system during the operation.

#### Neuroanesthesia

Standard monitoring included ECG, pulsoxymetry, noninvasive blood pressure, and was extended with invasive blood pressure, central venous line, capnometry, temperature-sensing urinary catheter and stomach tube. Neurosurgery was performed under general anesthesia with endotracheal intubation according to a standardized protocol, which was based on sufentanil (0,15–0,30 μg kg-^1^) and sevoflurane (1.5–2.0 vol%). In addition, patients received rocuronium (0,6–1,0 mg kg-^1^) to faciliate deep anesthesia. Along with MgSO_4_ 10%, intravenous esketamine was administered continuously at a rate of 0.1 mg kg-^1^ h^−1^ during surgery. All patients were ventilated controlled (Cicero EM, Perseus A 500, Dräger Medical Deutschland GmbH, Lübeck, Germany). Ventilation was set to keep an arterial carbon dioxide tension of 30-35 mmHg and whenever possible, a positive endexspiratory pressure was avoided. The core temperature was kept at 35–36 °C by Bear Hugger™ (3 M, St. Paul, MN, U.S.A.). Right before clipping the aneurysm, the mean arterial blood pressure was reduced in a controlled setting to 50-60 mmHg and simultaneously the FiO_2_ was increased to 50%.

#### Administration of magnesium sulfate

Magnesium sulfate MgSO_4_ 10% was administered after dura opening with 50 mg/kg body weight at a continuous infusion rate. The rate depended on the blood pressure (mean arterial pressure > 60-65 mmHg) and was administered over a maximum of 60 min. The dose was based on previous studies to rapidly evoke an effective magnesium concentration [21, 22]. Serum magnesium concentrations were measured 3 h after administration of MgSO_4_.

Patient data were automatically recorded and stored online. For further processing, relevant data were extracted from patient record using NarkoData (V.4.9.4.7917, latest system version update 27.04.2016, IMESO GmbH, Hüttenberg, Germany).

#### Postoperative care and follow-up

After surgery, patients were admitted to our ICU for 10 to 14 days. All patients were treated in accordance with the guidelines of our department. We recorded neurological outcome and clinical status using the modified Rankin Scale (mRS) at discharge from hospital and 12 months later. Outcome measures were surgery-related morbidity and mortality as well as appearance of neurological deficits as documented during hospital stay. Cerebral vasospasm was detected either by transcranial Doppler ultrasound using the Lindegaard ratio ≥ 3 and a mean flow velocity of ≥120 cm/s [23] or digital subtraction angiography based on the definition of Bradford and colleagues [24].

#### Statistical analysis

The SPSS Version 21.0 for Windows (SPSS Inc., Chicago, Illinois, U.S.A.) was used for statistical analysis. Results are given as median values and one standard deviation (SD). Dependent variables were “SO2”, “rHb”, “velo” and “flow”. Independent variables were the presence or absence (before/after) of MgSO_4_ administration. A Shapiro-Wilk-Test was performed to identify normal distribution for each continuous variable. We used a paired *t test* in cases with Gaussian distribution, otherwise the non-parametric *Wilcoxon signed-rank test* was used for paired comparisons (before/after) of independent variables. To identify possible confounding factors, we examined correlation of O2C parameters with the following anesthesiological parameters: bladder temperature, systolic/diastolic blood pressure, mean arterial blood pressure, heart rate, end-tidal partial carbon dioxide pressure (etCO_2_), temperature corrected partial carbon dioxide pressure (pCO_2_ (T)), fraction of inspired oxygen (FiO_2_), hemoglobin oxygen saturation (SaO_2_), hemoglobin (Hb), hematocrit (Hct) and arterial bicarbonate (HCO_3_). Here, Spearman’s rank correlation coefficient was calculated. Statistical significance was set at *P* < 0.05.

## Results

All recordings were performed successfully in every patient. No adverse events of MgSO_4_-administration were detected. All O2C parameters were not normally distributed. Cardiorespiratory and anesthesiological parameters were kept constant throughout the operation. The median time between onset of symptoms and application of MgSO_4_ was 2.8 (min. 1.6, max. 15.5) hours. Median serum magnesium levels were 1.3 ± 0.3 mmol/L.

**Fig. 2:**
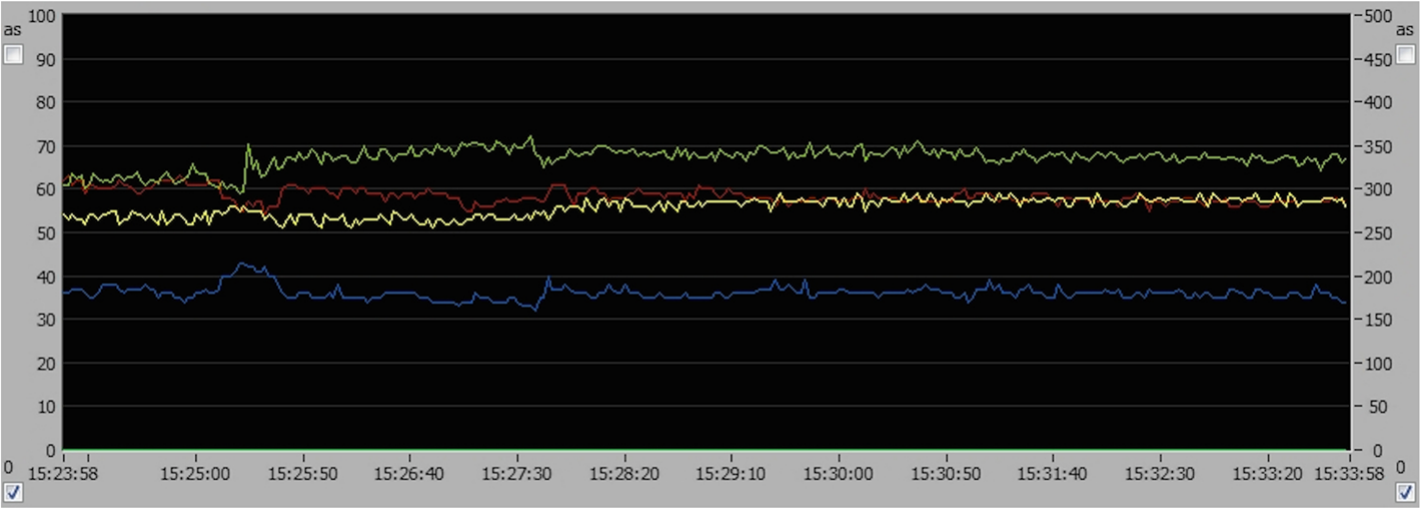
Time course of O2C-parameters during real-time monitoring in patient No. 13. Values are displayed in percent (%; left ordinate) or arbitrary units (AU; right ordinate) over time (hrs, min, sec; abscissa). After application of magnesium sulfate (baseline, 15:23:58), local cerebral blood flow (green) and blood cell velocity (yellow) gradually increase. Relative hemoglobin content (red) and capillary venous oxygenation (blue) tended to decrease over the displayed time period of 10 min

Statistical analysis revealed a significant increase of median flow from a baseline value of 313.6 ± 77.8 (min. 214.0, max. 479.5) to 375.3 ± 79.4 AU (min. 266.0, max. 565.0; *p* = .001), which was 20.8%. All other parameters showed no statistically significant differences. Blood cell velocity had a tendency to rise from 52.4 ± 10.7 AU (min. 32.0, max. 65.0) to 54.1 ± 11.4 AU (min. 33.0, max. 69.0; *p* = .10), which means a slight increase of 4.9%. SO2 decreased 9.4% by trend from 48.4% ± 16.9 (min. 22.0, max. 79.0) to 43.4% ± 14.0 (min. 19.0, max. 72.0; *p* = .16). Hemoglobin content (rHb) tended to decrease slightly by 1.5% (59.0 ± 16.6 AU, min. 34.0, max. 95.0 to 56.7 ± 13.8 AU, min. 30.0 max. 81.0; *p* = .55). A typical time course of MgSO_4_ application is given in Fig. 2.

The increase in blood cell velocity and blood flow indicates a rise in local blood perfusion. Although not statistically significant, capillary-venous oxygen saturation showed a tendency to decline, which could be an expression of a higher oxygen extraction rate. The trend of a decrease in relative hemoglobin content (rHb) as a surrogate marker for relative blood volume appears along with a higher blood flow, causing a larger shift of blood volume per time.

We identified two anesthesiological parameters that affected the O2C measurement values. We found a strong positive correlation between velo and FiO_2_ (r_s_ = 0.712, *p* = 0.004) and a strong negative correlation of SO2 with HCO_3_ (Spearman Rho coefficient r_s_ = −0.599, *p* = 0.024). An overview of anesthesiological values is given in Table 2.

**Table 2:** Anesthesiological parameters before (baseline) and after 10 min of MgSO4 application (10 min) given in median ± SD

	Baseline (0 min)	MgSO4 (10 min)	O2C parameter; Correlation coefficient
Temperature (°C)	35.4 ± 1.3	35.4 ± 1.5	
Hb (g/dl)	10.95 ± 1.6	11.3 ± 1.3	
HCO_3_^*^ (mmol/l)	23.65 ± 1.3	24.0 ± 1.4	SO2; r_s_ = −0.599, p = 0.024
Hct (%)	33.8 ± 4.8	34.3 ± 4.02	
pCO_2_ (T) (mm Hg)	37.6 ± 4.5	38.1 ± 4.7	
FiO_2_^*^	0.5 ± 0.1	0.5 ± 0.1	velo; r_s_ = 0.712, p = 0.004
SaO_2_ (%)	99.1 ± 1.5	99.0 ± 0.8	
etCO_2_ (mm Hg)	30.5 ± 3.4	30.5 ± 2.8	
TcpO_2_	100.0 ± 2.6	100 ± 1.0	
BP_sys (mm Hg)	100.5 ± 11.7	102 ± 8.7	
BP_dia (mm Hg)	53.5 ± 7.0	52.5 ± 6.7	
MAP (mm Hg)	69.0 ± 8.4	71.5 ± 7.5	
HR (bpm)	65 ± 14.1	64.5 ± 13.3	

*Hb* hemoglobin, *HCO*_*3*_ bicarbonate, *Hct* hematocrit, *pCO*_*2*_ *(T)* temperature corrected partial carbon dioxide pressure, *FiO*_*2*_ fraction of inspired oxygen, *SaO*_*2*_ hemoglobin oxygen saturation, *etCO*_*2*_ end-tidal partial carbon dioxide pressure, *TcpO*_*2*_ transcutaneous tissue oxygen tension, *BP_sys* systolic blood pressure, *BP_dia* diastolic blood pressure, *MAP* mean arterial blood pressure, *HR* heart rate; *p < .05, correlation analysis using Spearman’s rank correlation coefficient (r_s_)

In two patients, SEP monitoring was not possible due to a high impendance and internal system measurement error, most likely related to the skin flap size and retraction. In 8 of 12 patients, pathological SEP values correlated to an ischemia-related post-operative neurological deficit.

Cerebral vasospasm was detected in 8/14 patients, however, only two out of these eight patients suffered from symptomatic vasospasm with an apparent ischemia as documented by CT and the corresponding neurological deficit. Thus, the rate of clinical relevant vasospasm causing a delayed cerebral ischemia was 2/14 (14%) in our patient group (Table 1).

Of fourteen patients included in this study, two patients (No. 9 and 12) succumbed to the severity of their SAH, which caused multiple infarctions, malignant brain edema and ultimately, transtentorial herniation of brain tissue leading to central dysregulation and death during their hospital stay. Complications occurred in 3 patients (21%). Two patients suffered from cerebral infarctions due to surgical manipulation of the recurrent artery of Heubner and perforators (No. 1 and 5). Patient No. 10 had a brain abscess, which was evacuated 23 days after clipping and treated with an abscess drainage and i.v. antibiotics.

## Discussion

Based on our clinical observations over the past years, we wanted to test the hypothesis that early administration of magnesium sulfate increases local cerebral microcirculation in patients with SAH in this preliminary study. To achieve this, we used a novel combined laser-Doppler flowmetry and tissue spectrophotometry system. The intraoperative non-invasive real-time measurement of microcirculatory parameters revealed an increase in blood flow and blood cell velocity with a tendency of increase in capillary-venous oxygen saturation and decrease in the capillary filling pressures, indicating a higher local cerebral blood flow. This phenomenon was observed approximately 10–15 min after the early intravenous administration of MgSO_4_. Analysis of possible confounding factors revealed a strong positive correlation between velo and FiO_2_, which is probably due to the measurement method of the O2C-System. One explanation of the negative correlation seen in SO2 and HCO_3_ might be that a reduction in capillary-venous oxygen saturation is associated with a reduction in partial venous oxygen pressure. This reduction could be part of the oxidative metabolism, which causes an increase of carbon dioxide. The latter is buffered by the carbonic acid – bicarbonate system and consequently, a higher HCO_3_ concentration is found.

Magnesium sulfate has neuroprotective properties such as relaxation of cerebral arteries and arterioles in a concentration-dependent manner. Studies suggest that it is capable to reverse postischemic cerebral vasospasm, and offers neuronal protection through direct metabolic depression of glucose, or blocking of excitatory neurotransmitters [25, 26]. Magnesium antagonizes the function of calcium by competing for the N-methyl-D-aspartate (NMDA) receptors, which leads to an inhibition of postsynaptic potentials [1]. Furthermore, magnesium appears to suppress the formation of free radicals after tissue injury [27, 28]. Althogether, these effects are accounted for protection of injured brain tissue by preventing the occurrence of a postischemic excitotoxic phase [1, 25].

Drawbacks of the application of magnesium in humans are e.g. hypotension, bradycardia, loss of reflexes or respiratory depression [1, 16, 29, 30]. The most serious side effects of magnesium result from its antagonistic action on the calcium ion. Therefore, magnesium should not be given in case of hypotension or severe atrioventricular block. In addition, magnesium dosage should be reduced in patients with renal failure.

Magnesium can cross the blood brain barrier in humans and animals, however, serum levels cannot reliably be compared to the concentration of magnesium in the cerebrospinal fluid.

Although there are several studies that suggest a neuroprotective effect during neurosurgical procedures associated with ischemia [14], three large RCTs found no statistically significant benefit regarding the neurological outcome after application of magnesium in stroke patients.

There are several possible reasons for those results. First, comparisons in meta-analysis are confounded by the heterogeneity in methodology and patient groups. Examples are 1) initiation and timing of magnesium administration, 2) dose and concentration levels, 3) duration of administration, 4) no study information about the clinical correlation of hypoxemia, morphological changes in MRI/CT and angiographic vasospasm. The mean time from symptom onset to initiation of therapy was 31.7 h in IMASH and 33 h in MASH-2. In our opinion, the time of administration of MgSO_4_ is crucial, as the acute phase of SAH determines subsequent pathophysiological mechanisms such as early brain injury and the extent of cerebral vasospasm or delayed ischemic deficits at a later point in time (see below). Moreover, serum magnesium levels were not measured as study parameters in MASH-2, making comparison to the other RCTs difficult [14]. The magnesium sulfate dose in the MASH and MASH II trials was continuous infusion of 64 mmol/L per day for the duration of up to 18 days (MASH) or 20 days (MASH II) with maintaining serum magnesium levels within 1.0 to 2.0 mmol/L. In IMASH, 20 mmol MgSO4 was administered over 30 min followed by a continuous infusion of 80 mmol per day for up to 14 days after hemorrhage. Infusion rate was adjusted to a magnesium plasma concentration twice the baseline value and below 2.5 mmol/L. Dose-dependent effects and signs of hypermagnesemia are reported with serum magnesium levels above 2.0 mmol/L, which include headache, muscle weakness and nausea. Magnesium levels between 2.2 and 3.1 mmol/L can induce bradycardia, hypertension, and bradypnea [21].

In our patient group, MgSO_4_ was applied within a median time span of about 3 h after onset of symptoms. We regulated the infusion rate based on the MAP to maintain stable cerebral perfusion pressures. As the semiquantitative variables of the O2C system are not validated by invasive measurements of cerebral blood flow in this study, the relative increase in local cerebral blood flow cannot be depicted in absolute values. Even though we observed a delayed cerebral ischemia in 14% of our cases, and this rate is below the reported incidence of 30–40% in SAH patients, our vasospasm rate was 50% [31]. Therefore, a direct implication for a significant reduction of DCI or cerebral vasospasm cannot be drawn due to the small sample size and lack of randomization.

Another important factor of this study is the comedication of esketamine in all of our patients. As an N-methyl D-aspartate (NMDA) receptor antagonist, ketamine is known to have a vasodilatory effect in cerebral blood vessels and can increase regional and global cerebral blood flow alike. There is evidence that this vasodilatation is calcium-mediated [32]. As MgSO_4_ acts as a non-competitive inhibitor of the intracellular IP3-gate calcium channel and voltage-dependent calcium channels, it can intensify the vasodilatory effect of ketamine. Moreover, ketamine protects brain tissue by maintaining intracellular magnesium levels due to NMDA and quisqualate receptor blockade [33, 34].

The fact that augmentation of cerebral blood flow does not automatically convert into better clinical outcome of patients with post-SAH vasospasm has been under investigation since the results of the Clazosentan trials. Despite reversal of angiographic vasospasm after application of the selective endothelin(ET)-1 receptor antagonist, morbidities and functional neurological outcome remained unchanged [35]. Other pathophysiological mechanisms that have been addressed are early brain injury within 72 h after the bleed, which is believed to be a result of a peak in intracranial pressure and ischemia at the moment of the aneurysm rupture [8]. This incident can trigger a cascade of events including early activation of inflammatory processes, capillary thrombosis, blood-brain barrier dysfunction, cerebral edema, neuronal apoptosis and cortical spreading depression [36]. The latter describes a depolarization wave that spreads across the grey matter and results in depression of EEG activity. The incidence of cortical spreading depression seems to correlate with the point in time where DCI occurs in the early and late state after SAH [37]. The conclusion that could be drawn from the current data is that the pathophysiological mechanism of vasospasm and the post-SAH delayed ischemic deficit extends beyond changes in macrocirculation such as vasoconstriction, but to the level of cerebral microcirculation.

To the best of our knowledge, this work is the first study on the impact of early MgSO_4_ application on cerebral microcirculation using non-invasive vascular real-time monitoring in patients with aneurysmal SAH. The system used is a novelty, as is combines both techniques, laser-Doppler flowmetry and tissue spectrophotometry, in one device. Although there is one previous study investigating the feasibility of this method in intracranial aneurysm surgery, it did not handle the scientific subject of magnesium application and moreover, continuous real-time monitoring was not performed [18].

### Limitations of this study

The data of this study are preliminary without a control group receiving placebo instead of MgSO_4_ in a randomized, double-blinded design. Microcirculatory parameters as assessed by the O2C system are only of semiquantitative nature. Regarding the statistical calculation of O2C variables with a small sample size, an a priori power analysis could not be carried out due to the lack of reference values, which is another limiting factor of our study and the interpretation of results [38]. We did not determine the plasma or cerebrospinal fluid concentrations of magnesium regularly, however, in patients No. 12, 13 and 14, both blood and CSF magnesium levels increased by 30–40%. As a non-invasive neuromonitoring technique using light waves, measurement errors can occur due to ambient light or motion artifacts, or incorrect placement of the probe. Another drawback is the local, and not regional measurement volume, which covers only a few cm^3^. Even though the system has the advantage of recording blood flow as well as metabolism modalities, the interaction of pathophysiological mechanisms and the disturbance of the blood-brain barrier as well as cerebral autoregulation can be confounders to our results.

Future studies should deal with the exact concentration of MgSO4 in blood serum and CSF to determine the optimum rate and duration of early magnesium infusion for the prevention of cerebral vasospasm and the associated delayed neurological deficit. Non-invasive vascular neuromonitoring could be a useful tool to detect the impact on cerebral microcirculation to guide the application of MgSO_4_. A randomized, double-blinded controlled trial is needed for the proof of concept.

## Conclusions

The early intravenous application of magnesium sulfate in patients with aneurysmatic subarachnoid hemorrhage affects local cerebral microcirculation. Our preliminary data using non-invasive continuous real-time monitoring suggests an increased cerebral blood flow. Further trials are needed to underline the potential benefit of the point in time, dosage and application rate of magnesium as well as the vascular neuromonitoring to prevent vasospasm and subsequently, a delayed ischemic neurological deficit.

## Abbreviations

AU: Arbitrary units
CSF: Cerebrospinal fluid
CT: Computed tomography
CVS: Delayed cerebral vasospasm
ECG: Electrocardiogram
flow: Blood flow
MgSO_4_: Magnesiumsulfate
NMDA: N-methyl D-aspartate
RCT: Randomized controlled trial
rHb: Relative hemoglobin content
SAH: Subarachnoid hemorrhage
SEP: Somatosensory evoked potential
SO2: Capillary-venous oxygen saturation
velo: Blood cell velocity
WFNS: World Federation of Neurosurgical Societies
